# Electrospinning Nanofibers for Therapeutics Delivery

**DOI:** 10.3390/nano9040532

**Published:** 2019-04-03

**Authors:** S. M. Shatil Shahriar, Jagannath Mondal, Mohammad Nazmul Hasan, Vishnu Revuri, Dong Yun Lee, Yong-Kyu Lee

**Affiliations:** 1 Department of Chemical and Biological Engineering, Korea National University of Transportation, Chungju 27469, Korea; shatilshahriar26@gmail.com; 2 Department of Green Bio Engineering, Korea National University of Transportation, Chungju 27469, Korea; jagannathrkm@gmail.com (J.M.); nazmulchemi@gmail.com (M.N.H.); vishnurevuri91@gmail.com (V.R.); 3 Department of Bioengineering, College of Engineering, and BK21 PLUS Future Biopharmaceutical Human Resources Training and Research Team, and Institute of Nano Science & Technology (INST), Hanyang University, Seoul 04763, Korea

**Keywords:** electrospinning, nanofibers, fabrication, therapeutics, biomedical applications

## Abstract

The limitations of conventional therapeutic drugs necessitate the importance of developing novel therapeutics to treat diverse diseases. Conventional drugs have poor blood circulation time and are not stable or compatible with the biological system. Nanomaterials, with their exceptional structural properties, have gained significance as promising materials for the development of novel therapeutics. Nanofibers with unique physiochemical and biological properties have gained significant attention in the field of health care and biomedical research. The choice of a wide variety of materials for nanofiber fabrication, along with the release of therapeutic payload in sustained and controlled release patterns, make nanofibers an ideal material for drug delivery research. Electrospinning is the conventional method for fabricating nanofibers with different morphologies and is often used for the mass production of nanofibers. This review highlights the recent advancements in the use of nanofibers for the delivery of therapeutic drugs, nucleic acids and growth factors. A detailed mechanism for fabricating different types of nanofiber produced from electrospinning, and factors influencing nanofiber generation, are discussed. The insights from this review can provide a thorough understanding of the precise selection of materials used for fabricating nanofibers for specific therapeutic applications and also the importance of nanofibers for drug delivery applications.

## 1. Introduction

Nanotechnology—the use of nanomaterials for biomedical applications—is an emerging and promising paradigm in biomedical research. Nanomaterials with exceptional physiochemical properties, biocompatibility and minimal biological toxicity, can sense local biological environments and initiate cellular level reprogramming to achieve the desired therapeutic efficacy. Diverse zero dimensional (quantum dots, carbon dots, graphene quantum dots), one-dimensional (nanorods, nanowires, nanotubes, nanofibers) and two-dimensional (graphene oxide, transition metal dichalcogenides, transition metal oxide, MXens, etc.) nanomaterials along with nanosized particles are currently used for diagnosis, imaging and therapy [1,2,3]. Nanofibers are fiber shaped nanostructures, typically with two of their dimensions in nanoscale. Nanofibers have a high surface area-to-volume ratio with tunable porosity and can easily be functionalized with biological molecules. The choice of a wide variety of materials, such as natural and synthetic polymers, inorganic nanomaterials, composites and biomolecules as drugs for nanofiber fabrication makes them a robust and attractive candidate for many advanced biomedical applications [4,5,6,7,8,9,10,11,12]. These remarkable characteristics make nanofibers an ideal nanomaterial for energy generation and storage, water and environmental treatment, and healthcare and biomedical engineering applications.

Electrospinning is a unique and versatile technique that depends on the electrostatic repulsion between surface charges to constantly draw nanofibers from viscoelastic fluids. Polymers, ceramics, small molecules and their combinations are used as rich materials for the production of nanofibers. In addition to solid nanofibers, a secondary structure of nanofibers—including porous, hollow or core-sheath structures—has been manufactured and the surface of the structure can be functionalized with different molecular moieties, during or after the electrospinning process [13]. Electrospinning is the main method of choice for the large scale production of nanofibers due to its controllable diameter, easy handling, minimum consumption of solution and cost effectiveness. Electrospun nanofibers have various biomedical applications, such as wound dressings, drug and gene delivery tools, sensors and catalysts [14]. In this review, our focus is on addressing the application of electrospun nanofibers in therapeutics (drug/gene) delivery. Various therapeutics delivery systems have been investigated for efficient loading, releasing and accumulation of the therapeutics into the target site. However, electrospinning exhibits great flexibility for selecting diverse materials, drugs and genes (DNA, RNA etc.) for therapeutic applications [15].

We have reviewed the most efficient and recent therapeutic applications of electrospun based nanofibers and their future perspectives. Different electrospinning techniques are described here, along with their biomedical applications and suitability. Nanofibers are generally natural or synthetic polymers and they have vast therapeutic applications even though only a limited number reach clinical trials. The application of nanofibers as drug delivery carriers—especially for cancer drugs, antibacterial drugs, nonsteroidal anti-inflammatory drugs, cardiovascular agents, gastrointestinal drugs, antihistamine drugs, contraceptive drugs and palliative drugs delivery—is described here. Nanofibers have promising therapeutic applications for DNA, RNA and growth factors, for example, protein and steroid hormones delivery, which are included in this review. The current scenario for nanofiber based materials is mostly promising for wound dressing, sensor technology and for the catalysis of various reaction pathways in the laboratory [14]. The therapeutic application of nanofibers to drug or gene delivery is limited due to proper functionality, controlling capacity, toxicity and large-scale production limitations. However, the development of nanofiber based delivery systems is growing rapidly and many of them have shown excellent characteristics for future therapeutic delivery applications [15]. This review depicts the whole scenario around current therapeutic delivery systems based on nanofibers and their useful biomedical applications, as well as suggesting future perspectives for nanofiber based delivery systems, and provides relevant literature for researchers working in this area.

## 2. Electrospinning Techniques

**Figure 1:**
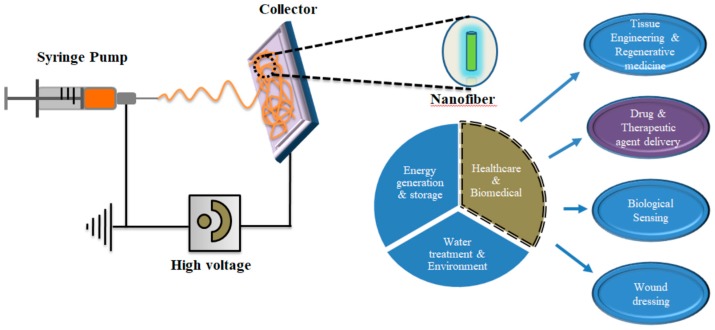
Schematic representation of a traditional electrospinning process and various healthcare and biomedical applications of nanofibers.

Electrospinning is considered a promising, highly productive and simple method for fabricating the nanofibers of polymers, composites, and inorganic materials, including carbides, oxides, nitrides and hybrid composites. In the electrospinning technique, electrostatic forces are utilized to produce nanofibers from a polymer solution [16]. In general, the electrospinning setup consists of three main compartments, namely: (i) high voltage power supply; (ii) a spinneret; and (iii) a conductive collector, as shown in Figure 1. In the electrospinning process, a potential (kV) is applied between the spinneret and the collector. These parts conduct electricity and are separated at an optimum distance. When the applied electric field overcomes the surface tension of the droplet, a charged jet of polymer solution can be ejected from the tip of the needle. The jet grows longer and thinner, with an increasing high diameter loop, which results in the solidification of the polymer due to solvent evaporation. The solidified nanofibers are then collected on the target. In general, electrospun nanofibers are categorized in two ways; namely, random and aligned nanofibers [16]. Random nanofibers can be produced using a simple plate collector, while aligned nanofiber mats or uni-axial fiber bundles can be produced using a disc or cylinder, rotating at a high speed, as the collector, along the direction of rotation. The unique physical characteristics of electrospun nanofibers, such as high surface-to-volume ratio, controllable fiber diameters and surface morphologies (dense, hollow, and porous) and fibrous structures, can be altered by modulating parameters, for example: (i) the molecular weight of polymers and polymer solution properties (viscosity, conductivity, dielectric constant, and surface tension) [17,18]; (ii) The processing parameters (such as the electric potential, flow rate, feeding rate, distance between the capillary and collection, as well as using coaxial or triaxial needles for hollow, core–shell or multi-sheathed structures); and (iii) controlled post processing parameters (such as heating rates and heating temperatures, especially for inorganic materials) [19].

## 3. Types of Electrospinning

Although fibers produced by different electrospinning methods have attracted increasing attention in the field of biomedical applications, challenges persist for the selection of an appropriate method, as well as for optimizing multiple parameters to generate robust cargo loaded nanofibers [20]. Electrospinning techniques can be classified into five categories: (i) blend electrospinning; (ii) coaxial electrospinning; (iii) emulsion electrospinning; (iv) melt electrospinning; and (v) gas jet electrospinning. Blend electrospinning can be used to develop nanofibers with burst release, while co-axial and emulsion electrospinning can be used to generate core-shell nanofibers that can assist in sustained drug release [21]. Melt electrospinning is a cleaner fabrication method that has been used extensively to produce highly ordered electrospun nanofibers. However, melt electrospinning results in the generation of fibers with a larger diameter [22]. The basic mechanism of each electrospinning setup and the process parameters affecting the fiber morphology are discussed below. 

### 3.1. Blend Electrospinning

Blend electrospinning is the conventional electrospinning technique, in which the drug/biomolecules are dispersed or dissolved in the polymer solution, resulting in the development of nanofibers with drugs/biomolecules dispersed throughout the fibers [23]. Although this method is simple in contrast with other electrospinning methods, the solvents used for the dispersion of bioactive molecules can lead to protein denaturation or loss of biological activity. In addition, the inherent charge of the biomolecules can often result in their migration on the jet surface and thereby result in their distribution on the surface of the nanofibers rather than the encapsulation of biomaterials within the fibers. The surface dispersion can be associated with the burst release of the biomolecules [24].

### 3.2. Co-Axial Electrospinning

Co-axial electrospinning is an improvement on conventional blend electrospinning, in which two nozzles are connected to the high voltage source rather than one nozzle. Two different solutions are loaded within each nozzle and are pumped out to generate nanofibers with core-shell morphologies [25]. Both synthetic and natural polymers can be used to develop core-shell nanofibers with improved physiochemical and biological properties. This method is advantageous over the blend electrospinning method, as they can protect as well as overcome the denaturation of drugs/biomolecules in the biological system [26]. In co-axial electrospinning, the biomolecules or drugs are situated in the inner jet and are co-spun with the polymers present in the outer jet. This results in protection of the cargo, as well as assisting their being sustained in the biological environments [27,28]. For example, Merkle et al. used polyvinyl alcohol (PVA) and gelatin for the fabrication of core-shell nanofibers [29]. The mechanical strength of the core PVA phase was improved by increasing the amount of gelatin in the shell. The authors have claimed that the gelatin shell augmented cell adhesion and fibroblast adhesion onto the surface of the PVA/gelatin fibers, compared to the PVA fibers. In addition, coaxial fibers can be surface-modified with biomolecules to improve their biofunctionality and enhance cell-surface interactions [30]. Apart from the design complexity, the viscoelasticity and interfacial tension of the core and shell polymers must be thoroughly monitored to generate core-shell nanofibers from co-axial electrospinning. 

### 3.3. Emulsion Electrospinning

Emulsion electrospinning requires a setup similar to that of blend electrospinning, in which two immiscible solvents are simultaneously spun to generate core-shell nanofibers. Here, active bioactive molecules, along with surfactants, are initially allowed to form W/O emulsions and are later mixed with the polymer matrix solution [25]. During the fiber trajectory, the emulsion droplets are stretched into elliptical shape. Furthermore, the continuous phase solvent is swiftly evaporated, resulting in the viscosity gradient as well as droplet enrichment in the axial region [31]. This viscosity gradient between the polymer matrix and elliptical droplet guides the core material to settle within the fiber matrix rather than on the polymer surface. This method is relatively simple compared to co-axial electrospinning and also offers sustained release of the loaded cargo materials [32]. Applied voltage has a significant effect on the nanofiber diameters. Higher applied voltage ensues in the generation of nanofibers with a reduced diameter. In addition, other parameters like flowrate and spinning distance can also affect fiber morphology [33]. However, the interface tension between the organic and aqueous phases of the emulsion can damage the bioactive molecules loaded within the emulsion electrospun fibers [34]. 

### 3.4. Melt Electrospinning

Melt electrospinning has gained more attention in the electrospinning field, where toxicity and solvent accumulation is a concern. In this process, polymer melt is used instead of a solution, which is transformed from liquid to solid to achieve the desired product on cooling, rather than through solvent evaporation [22]. The polymer melt flow rate and homogeneous polymer melt conditions must be controlled to produce high quality fibers of uniform morphology, but with a broad range diameter. Polymer blends [35] and additives [36] have been used to reduce the average diameter of the fibers. The effect of the melt temperature can affect the structure and function of drugs, proteins and bioactive molecules loaded in the fibers [37]. The flow rate and melt viscosity of melt electrospinning can influence the characteristics of the resulting fibers. The surface wettability of melt electrospun fibers has improved through the formation of hydroxyl or peroxyl and N-containing functional groups, respectively, by using oxygen and ammonia plasma [35,38]. 

### 3.5. Gas Jet Electrospinning

Gas jet electrospinning is an improvement on the conventional melt electrospinning technique, in which the conventional electrospinning setup is additionally equipped with a gas jet device. The major limitation of melt electrospinning is that it requires definite control over the temperatures and therefore multiple heating zones must be placed to maintain the polymer melt. This results in generation of thicker nanofibers compared to the solutions. In this technique, the co-axial jet is surrounded by a tube feeding the heated gas, which can provide sufficient heat near the nozzle and delay the process of polymer solidification. For example, Zhmayev et al. spun polylactic acid (PLA) and showed a decrease in the diameter of the nanofibers obtained by gas jet electrospinning compared to normal electrospinning. Interestingly, the heated gas flow-rate had a significant effect on the diameter of the nanofiber. The increased gas flow rate can offer additional drag force to the jet surface which can result in the development of thinner nanofibers. Zhmayev et al.’s result showed a significant decrease in the diameter of the nanofiber, from 350 nm to 183 nm, when the gas flow rate was increased from 5.0 L/m to 15.0 L/m [39,40].

## 4. Therapeutics Delivery Systems

**Figure 2:**
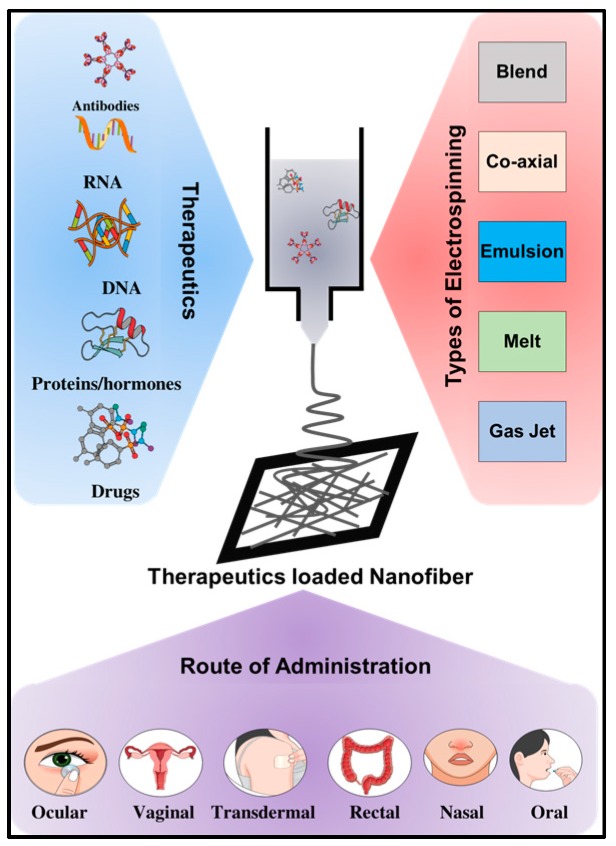
Types of electrospinning, different therapeutics-loaded nanofibers and their route of administrations.

Most conventional drugs are hydrophobic and suffer from poor bio-distribution, solubility and stability in the biological system. Moreover, these drugs do not have the desired active targeting capabilities, which can result in non-specific systemic toxicity or faster elimination from the body, without achieving the desired therapeutic efficacy. Drug delivery systems (DDS) are approaches, formulations and technologies for transporting therapeutic agents to the targeted therapeutic site in the body [41]. The developed DDS technologies not only encapsulate the target drug/biomolecule but also attune their absorption, distribution, release and elimination with higher loading efficacy and safety. The drug release from DDS carriers depends on diffusion, degradation, swelling and affinity-based mechanisms [42]. As mentioned above, electrospun nanofibers are gaining significant attention as promising therapeutic nanocarriers. In addition, their impressive characteristics, including biocompatibility, biodegradability, and high therapeutic payload capacity, meet the prerequisites for a good therapeutic delivery candidate [13]. Employing electrospun nanofiber scaffolds as a therapeutic nanocarrier, different routes of administration (ROA) are being investigated. Drugs/therapeutics can be administered to any region/organ in the body by way of common routes, such as oral, parenteral (subcutaneous, intramuscular, intravenous, and intrathecal), sublingual/buccal, rectal, vaginal, ocular, nasal, inhalation, and transdermal, using electrospun nanofibers (Figure 2). Here, we present the common routes of drug administration by electrospun nanofiber [43]. 

### 4.1. Oral

Of all delivery routes, the oral route is considered the most preferable and convenient route of administration, which can overcome the problems associated with other routes of administration [44]. However, to achieve successful administration of therapeutics targeting the oral route of administration is a difficult task. Before designing a successful oral delivery system, scientists should consider the major key challenges, including the presence of acidic gastric juice in the stomach, along with proteases, mucosal barriers and intestinal retention, which can hamper the absorption of drug delivery systems into the body [45]. Electrospun nanofiber scaffolds offer a great opportunity to load and deliver both micro and macromolecules, targeting the oral route [46]. Some of the common features of electrospun nanofiber in oral drug delivery systems include targeted delivery of therapeutics, sustained release properties, high transfection efficiency, rapid onset of action and fascinating pharmacokinetic profiles. Another promising advantage of using electrospun nanofibers is that researchers can design any desirable release properties such as fast [47], controlled [48], biphasic [49] or delayed [50] releases of the drug. Various polymers are used for designing oral drug delivery systems utilizing electrospun nanofibers such as poly (lactic-co-glycolic acid) (PLGA), polyvinylpyrrolidone (PVP), poly(ethyleneoxide) (PEO), PVP/cyclodextrin, polyvinyl alcohol (PVA), polycaprolactone (PCL), PVP/ethyl cellulose, PVP/zein, Cellulose acetate, Eudragit L, hydroxypropyl methylcellulose (HPMC), Eudragit S, Eudragit S/Eudragit RS, and shellac [50]. 

### 4.2. Sublingual/Buccal

The oral mucosa has its own subdivisions according to its different regions, namely, buccal, sublingual and gingival. A few years ago, researchers were also interested in designing nanofiber based drug delivery systems for sublingual (under the tongue) or buccal (between the gums and teeth) routes of administration. The sublingual or buccal delivery systems usually allow the drug-loaded electrospun nanofibers to dissolve in the presence of mucus so that the drug can directly penetrate into the small blood vessels. Interestingly, these oromucosal routes of administration are the most studied sites for nanofiber-based therapeutics, offering versatile and multifunctional drugs, DNA, RNA, protein, peptide, growth factors or vaccine delivery platforms [51]. 

### 4.3. Rectal

Pediatric patients (aged under 6 months) usually cannot swallow any drug or food supplement. Under such circumstances, the rectal route could be the best alternative to oral drug delivery systems. In addition, the rectal route is also effective in cases of unconscious or vomiting patients. An electrospun nanofiber-based rectal drug delivery system is continually gaining popularity. In the treatment of post-operative peritoneal effusion following rectal/pelvic surgery, electrospun nanofibers could be considered a safe potential biocompatible, and biodegradable sealing fiber. The first clinical trial on the safety and sealing properties of electrospun nanofibers in lymphorrhea following pelvic surgery was published in 2014 [52]. The authors used a synthetic material (PuraMatrix) that consists of sixteen amino acid peptides to fabricate self-assembled nanofibers. A total of 20 colorectal cancer patients participated in the clinical trial. After a 2–3 month follow-up period, a significant reduction in post-operative drainage volumes was apparent in the experimental group compared with the control group. In another study, Modgill and co-workers investigated the permeability of penicillin from an extremely thin nanofiber scaffold through different biological membranes. The authors used PVA to fabricate ciprofloxacin-loaded ultra-thin nanofibers. In vitro permeability studies exhibited the potency of electrospun nanofibers compared with the plain drug. The PVA nanofibers revealed the highest ciprofloxacin permeability in the rectal mucosal membrane. The drug release study showed the controlled release behavior of ciprofloxacin from nanofibers in the rectal mucosal membrane, whereas the control group showed a high degree of fluctuations [53]. 

### 4.4. Vaginal

Electrospun nanofiber scaffolds targeting vaginal routes have recently been investigated. However, the acidic condition of vaginal mucosa (~pH 4) should be considered before designing vaginal drug delivery systems using nanofibers. For example, Brako et al. fabricated progesterone-encapsulated nanofibers targeting the vaginal route of administration. The authors used a mucoadhesive molecule (carboxymethylcellulose) for the fabrication of electrospun nanofibers. As-synthesized progesterone/carboxymethylcellulose electrospun nanofibers exhibited sustained release properties [54,55]. Another example of nanofiber-based vaginal drug delivery would be a study involving the anti-HIV drug (maraviroc), in which the authors spun maraviroc either with PVP or PEO and demonstrated fast drug dissolving properties upon contact with moisture [56]. 

### 4.5. Nasal

Currently, supramolecular peptides are used to fabricate electrospun nanofiber scaffolds. Electrospun supramolecular peptide nanofibers have proven their potential for intranasal vaccine delivery. For example, Si and coworkers developed intranasal administrated influenza vaccine using peptide nanofibers consisting of virus polymerase. This self-assembled nanofiber could elicit both humoral and cell mediated immune responses against the influenza virus. To be specific, nasal administrated nanofiber vaccine particles were initially ingested by antigen presenting cells in lung-draining mediastinal lymph nodes and activated both Th1 Th2 to produce the antibody against the given antigen. Furthermore, a high immune response was elicited without any use of vaccine adjuvant [57].

### 4.6. Ocular

Generally, various drops or ointments are used for ocular infection or inflammation. In these cases, those drugs are easily excreted from the eye very quickly. Therefore, to improve therapeutic efficacy and overcome the repetitive administration of an established ocular dosage, nanofiber-based ocular inserts have been considered and compared with eye drops or similar methods of dosage. Drug-loaded nanofiber scaffolds that allow the controlled release of incorporated drugs can be placed into the ocular mucosa. For example, the use of brimonidine tartrate (BT)-loaded electrospun nanofibers as ocular inserts were used to treat ocular infection and inflammation, which is described in detail in Section 5.1.9 [58]. 

### 4.7. Transdermal

Transdermal drug delivery systems (TDDS) deliver a drug locally, avoiding undesired drug distribution and possessing excellent skin permeability. Due to high solubility, great morphology and sustained drug release kinetics, electrospun nanofibers have been developed into TDDS. It has been reported that electrospun nanofibers could enhance solubility and form a transdermal patch for the biopharmaceutics classification systems II drugs with a high therapeutic efficiency and no cytotoxicity. Hydrophilic polymers with great permeation are the strength of TDDS, and several in vitro and in vivo evaluations suggest the possibility of using drug-loaded electrospun nanofibers in establishing TDDS [59].

## 5. Applications of Nanofibers in Therapeutics Delivery

Producing nanofibers with excellent physico-biochemical characteristics using an electrostatic spinning technique is considered to be an efficient multipurpose drug delivery strategy for delivering various therapeutic agents ranging from nanomedicines to macromolecules, including proteins and nucleic acids. Nanofibers can overcome several key challenges such as low solubility, loading efficiency, short circulation and the plasma half-life of drugs and are effective at improving the bioavailability of drug growth factors, DNA, RNA, and so forth. [60]. Using various biocompatible and biodegradable polymers (natural and synthetic or their hybrid combinations) in nanofiber production may overcome problems relating to the necessity of a second surgery to remove implants. In this section, we review the biomedical applications of electrospun nanofibers and delineate novel strategies employed to deliver drugs, biomolecules and nucleic acids by way of the electrospun nanofibers. 

### 5.1. Nanofibers in Drug Delivery

#### 5.1.1. Anticancer Agents

Discovering a new potential treatment strategy against cancer is very difficult due to several drawbacks of anticancer therapeutics such as imperfect solubility, impermanence in the circulatory bloodstream, poor accumulation in cancer cells, highly toxic to normal cells, low working efficiency in solid tumors and excess elimination profile [61]. To surpass such limitations, the nanofiber-based targeted drug delivery of anticancer therapeutics could be a promising strategy in the field of cancer nanomedicine. Electrospun nanofiber scaffolds have superior drug loading and transferring capabilities which not only amplify the therapeutic efficacy and potency of loaded drugs but also decrease undesirable side effects by ensuring high cellular accumulation of loaded drugs at the target site. These versatile drug carriers have proven to overcome the major limitations of traditional drug carriers that face anticancer drugs delivery, including poor therapeutic-loading capacity, non-targeted delivery, objectionable drug release and so on [62]. In addition, their sustained release properties downregulate the repetitive administration of drugs, thus improving patient compliance. 

**Figure 3:**
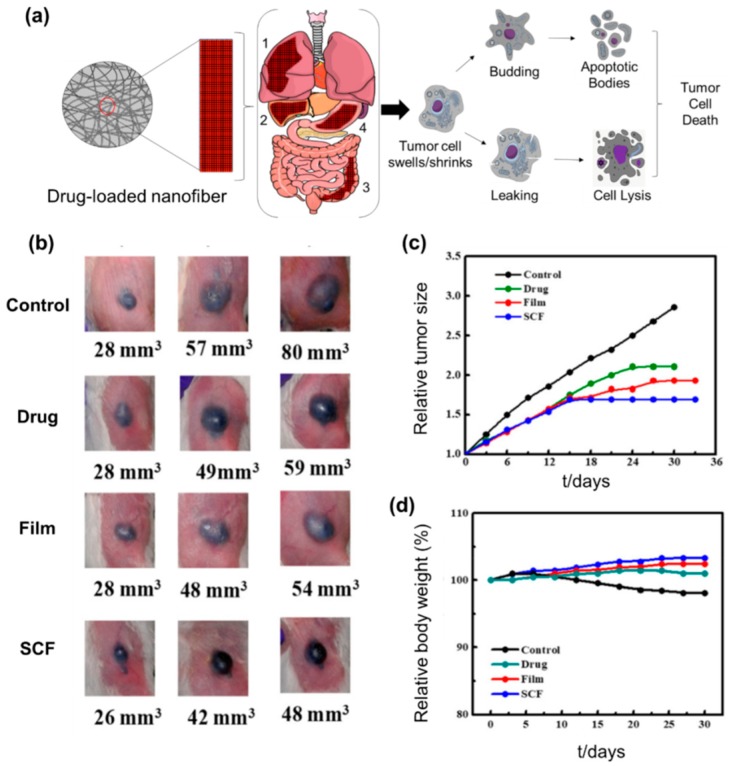
(**a**) The cytotoxicity effects of anticancer drug-loaded electrospun nanofibers targeting various deadly cancers. (Here, the numerical numbers 1, 2, 3, and 4 on the diagram represent the usages of nanofibers in lung, liver, gastric and colon cancer respectively.); (**b**) tumor dimension after treating with indicated patches (DXM-PLA); (**c**) quantitative tumor volume of mice treated with indicated scaffold/film/drug/control; (**d**) change of body weight of mice as a function of time upon treatment with indicated patch (Figure 3b–d reproduced from ref. [65] with permission from ACS Publication, 2019).

Recently, electrospun nanofibers have been widely used to ameliorate the solubility of anticancer drugs (Figure 3a). In this system, the drug-loaded nanofibers can circumscribe and crystallize the anticancer agents within the fiber, resulting in improvement of their dissolution rate in the biological system. For instance, water-insoluble anti-cancer drug Paclitaxel was loaded with surface modified mesoporous hollow stannic oxide nanofiber (SFNFP) in order to study whether electrospun nanofibers can improve the dissolution rate of Paclitaxel. The in vitro dissolution study results show that SFNFP exhibited an 8.34 times higher dissolution rate compared to naked Paclitaxel, over a period of 5 minutes. While the cumulative release rate of pure Paclitaxel was only 16.77 ± 2.00% after 1h, a high dissolution rate of 80.00 ± 2.64% was observed from SFNFP [63]. The release profiles of Paclitaxel from the SFNFP followed Noyes-Whitney’s drug release profiles. Extreme-sleazy shells functionalized nanofibers as core-shell structure were synthesized using the coaxial electrospinning method to load and enhance the dissolution rate of water-insoluble drugs Quercetin and/or Tamoxifen Citrate, as another example. The core of the nanofibers was composed of PVP-K90 or PCL while Quercetin/Tamoxifen Citrate along with surfactant SDS and PVP-10 as hydrophilic moieties were used to form the shell. The in vitro results suggested a faster release of insoluble drugs from nanofibers in dissolution media within a 1 min period. To be specific, 16.14% and 15.15% of pristine Quercetin and TC were released in 0.5 h respectively, while drug loaded nanofibers released Quercetin/TC either immediately or in 1 min [64]. The reason for a fast release could be due to the uniform distribution of the model drug in the extremely thin (100 nm in diameter) outer layer of the core-shell nanofiber scaffolds, allowing a large contact surface area and a short diffusion distance. Therefore, multifunctional nanofibers are able to dramatically increase the release profile of poorly water-soluble anticancer drugs regardless of the drug’s characteristics.

Even though polymeric nanoparticles (NPs) are used as a drug carrier to load anticancer drugs like Paclitaxel, the poor drug-loading efficiency of these systems minimizes the applications in the biological system [66]. On the other hand, nanofiber scaffolds using electrospinning facilitate higher drug loading because of large space and stereological honeycombed composition. Moreover, the choice of fabrication method for developing electrospun nanofibers, such as the coaxial process, emulsion methods, surface modification and blending, can tune/regulate the drug loading capacities of the nanofibers. For example, Xu and co-workers fabricated self assembleed Paclitaxel- Succinic Acid (PTX-SA) conjugate into supramolecular nanofibers. The loading efficiency of PTX in the as-synthesized nanoconstruct was more than 89%, which is considered the highest loading efficiency of PTX ever reported [67]. In addition, the controlled release of PTX from this nanoconjugate inhibited the proliferation of human lung adenocarcinoma cells in both in vitro and in vivo animal models. Compared to the burst release, the sustained release properties of cancer drug-loaded nanofiber scaffolds has great cytotoxic effects on tumor tissues. To understand these findings, Kumar et al. (2019) fabricated the anticancer drug dexamethasone (DXM)-loaded PLA nanofibers (DXM-PLA) in the form of a patch and applied it to a melanoma tumor in an experimental mice model. The sustained release properties of DXM from the as-synthesized nanopatch led to cytotoxic effects on cancer cells up to 85% within 3 days. Compared to the control groups, which resulted in 300% tumor growth, the DXM-PLA significantly prevented melanoma tumor cell proliferation and maintained the bodyweights of the animals (Figure 3b–d) [65]. 

Various reported scientific validations narrate that the circulation and plasma half-life of a drug nanocarrier highly depends on its size and shape. Geng et al. showed that uniquely shaped filomicelles like nanofibers can circulate in biological conditions for up to 7 days, which was 10 times greater than any type of polymeric nanoparticles, with which the drug can be eliminated from the bloodstream within 2 days. In addition, these filomicelles can selectively deliver the anticancer agent to the tumor tissues [68]. Furthermore, a versatile and scalable electrostatic spinning technique was employed to fabricate fiber rods with various sizes and shapes in order to confirm whether the shape and size of nanofibers can enhance the half-life, cancer tissue accumulation, cellular uptake, and tumor toxicity profiles of the anitcancer drug-loaded fiber rods. In this study, the electrospun fibers were treated under ultrasonication to fabricate fiber rods, where the lengths of the fiber rods were regulated by adding different volumes of sodium chloride (NaCl) void-precursors to the electrospun scaffolds. The experimental results revealed that when anticancer drug Doxorubicin was incorporated into fiber rods using PELA (RD_DOX_, 500 nm) and these nanocomposites were injected into a tumor-bearing mice model, it showed 4 times more accumulation in the cancer site and was 3 times more stable in plasma level compared with microspheres. However, small length nanofibers of 2 micrometers in diameter exhibited the most powerful cancer cell apoptosis and necrosis activities with the highest resistance to metastasis [69]. 

To reduce the undesirable side effects and toxicity of anticancer drugs for normal tissues, electrospun nanofibers are considered for targeted and pH-mediated drug delivery to tumors. The surface modification of electrospun nanofibers with ligands that can target specific receptors overexpressed on the tumor, along with pH-dependent tunable drug release, can be optimal for targeted drug delivery. For example, a chitosan/PLA solution was used to encapsulate graphene oxide (GO), titanium dioxide (TiO_2_) and a chemotherapy medication doxorubicin (Dox, C_27_H_29_NO_11_) into nanocomplexes through electrospinning. As-prepared chitosan/PLA/GO/TiO_2_/Dox fiber scaffolds showed an increased release of Dox in the acidic pH of a tumor microenvironment rather than in the physiological pH of 7.4 during a 200 h experiment. This characteristic would be imposed due to protonation of –NH_2_ in Doxorubicin that can disintegrate the –H bond between doxorubicin and nanofibrous scaffolds resulting in higher drug release in cancerous tissues [70]. The in vitro cytotoxicity studies revealed that chitosan/PLA/GO/TiO_2_/Dox fibers were biocompatible and did not expose any toxicity to normal cell lines. This research also evidenced that the cytotoxicity of nanofibers to tumor cells depends on the concentration of nanofibers—a higher concentration of nanofibers can increase both targetability and cytotoxicity to cancerous cells. A higher concentration of doxorubicin even appeared in the presence of an external magnetic field. As mentioned earlier, electrospun nanofibers can be developed with negligible toxicity towards normal cells by actively targeting the cancer cells. Heat shock protein (HSP 90) is highly overexpressed on lung cancer cell lines in humans. Researchers have developed novel stratgies by targeting HSP 90 to deliver anticancer drugs into tumor tissues of the lung [71]. Drugs like 17-DMAG (17-dimethylaminoethylamino-17-dimethoxy geldanamycin) can target both cancerous cells and the ATP-binding site of HSP 90, thus increasing the inhibition of both cancer cell proliferation in the lungs, and chaperoning the activities of HSP 90 and telomerase activity, respectively. Nevertheless, the unwanted side effects and extreme hepatotoxicity had minimized the use of 17-DMAG as a novel anticancer drug in lung cancer treatment [72]. To overcome such major problems, Mellatyar, and co-workers designed and developed 17-DMAG encapsulated PCL/PEG nanofibers via an electrospinning process. Their drug release profile revealed that about 96% 17-DMAG can release from PCL/PEG/17-DMAG fiber scaffolds after 6 h. In addition, the IC_50_ values and MTT assay results proved the potency of PCL/PEG/17-DMAG nanofibers over 17-DMAG in A549 cells cytotoxicity. After 3 days, the free 17-DMAG can reduce the HSP 90 expression level and telomerase activity up to 48% and 71% respectively whereas the percentages of inhibition by PCL/PEG/17-DMAG nanofibers were 79% and 83%, respectively [73]. These promising characteristics of PCL/PEG/17-DMAG nanofibers are connected with its controlled release properties over an extended period of time. Nevertheless, nanofiber scaffolds are able to easily target and enter into cancer cells due to the irregular vascular composition of tumor tissues. 

#### 5.1.2. Antibiotics

Since Alexander Fleming discovered and developed a true antibiotic named penicillin, antibiotics have been the most commonly prescribed and used pharmaceutical agents to treat various bacterial infections. Despite all the favorable characteristics of antibiotics, their appropriate delivery routes, toxicological profile, poor water solubility and, most importantly, microbial resistance are major limitations to their therapeutic efficiency. Although several delivery approaches have been proposed over the past few decades, issues associated with poor antibiotic loading efficiency, systemic toxicity and drug release profile limited their translation into clinical settings. In this scenario, electrospun nanofibers are considered as an alternative for antibiotic delivery because the large surface area and tunable pore size offer maximum antibiotic loading capacity and encapsulation efficiency. In addition, the current generation of nano-based fibers can also regulate the sustained and controlled release activities of antibiotics, maximize the dissolution rate of poor water-soluble antimicrobial agents and minimize systemic toxicity Antibacterial drugs which are encapsulated into nanofibers usually exhibit antimicrobial actions by inhibiting cell wall synthesis, protein synthesis, DNA/RNA synthesis, mycolic-acid synthesis, and folic acid synthesis. Penicillin has been widely studied as a model antibiotic to load within nanofibers for testing antimicrobial actions. For instance, an aminopenicillin drug like amoxicillin (AMX) was initially loaded into nanomicelle as a hydrophobic antibacterial drug via a film dispersion hydration method and later coaxial electrospining was performed to load AMX-loaded nanomicelle into the core/shell nanofiber (AMX/NM/NF). Antibacterial assays showed that AMX/NM/NF can create an inhibition zone of 9.2 mm and 7.3 mm against *E. coli* and *S. aureus* respectively [74]. 

The first criteria for developing an effective delivery system is to ensure the excess release of the encapsulated drug into the physiological environment. Khorshidi et al. developed an electrospun scaffold for loading a second generation fluoroquinolone antibiotic (ciprofloxacin), which can result in ultrasound-assisted drug release. These alginate-emanated nanofibers revealed 3 times more drug release properties with ultrasonic stimuli at 15 W/cm^2^ intensity and endowed higher percentages of bacterial DNA synthesis inhibition in both in *E. coli* and *S. aureus* [75]. The in vitro and in vivo studies further suggested that electrospun nanofiber scaffolds can enhance both the bactericidal and bacteriostatic activities of antibiotics. The use of metal ions and compounds, nanoparticles and salts are receiving burgeoning interest as antimicrobial agents. For example, iron oxide, silver, titanium dioxide, and zinc oxide either alone or in combination with other salts or ions are used as a core for generating antimicrobial nanofibers with bactericidal and/or bacteriostatic action. Among them, silver is considered the most potent antimicrobial agent due to its unique property of accumulating on the microbial cell wall and assisting in the arrest of the cell cycle and the denaturation of bacterial DNA [76]. Recently, Jatoi and co-workers proposed a new method to evaluate the antibacterial activities of silver nanoparticles and titanium dioxide by preparing cellulose acetate nanofibers, where TiO_2_ was first bound with DOPA followed by the introduction of AgNPs to form TiO_2_/AgNP. Finally, a TiO_2_/AgNP-loaded cellulose acetate nanofiber scaffold (CA/TiO_2_/AgNP) was fabricated with the electrospinning method. Their antimicrobial assays demonstrated that the as-synthesized CA/TiO_2_/AgNP nanoparticles (10 wt.% of TiO_2_/AgNP) showed almost 100% antibacterial activities against both *E. coli* and *S. aureus* for up to 3 days. In addition, CA/TiO_2_/AgNP nanoparticles can overcome severe adverse effects (argyria or argyrosis) that are commonly associated with silver nanoparticles-based antimicrobial therapy [77]. Moreover, bacteria usually lose the integrity of their cell wall when they come into contact with highly charged nanofibers like cationic chitosan, thus resulting in cell lysis. This non-release antimicrobial system is a promising strategy because of their substantially prolonged activities outside of drug resistance [76].

**Figure 4:**
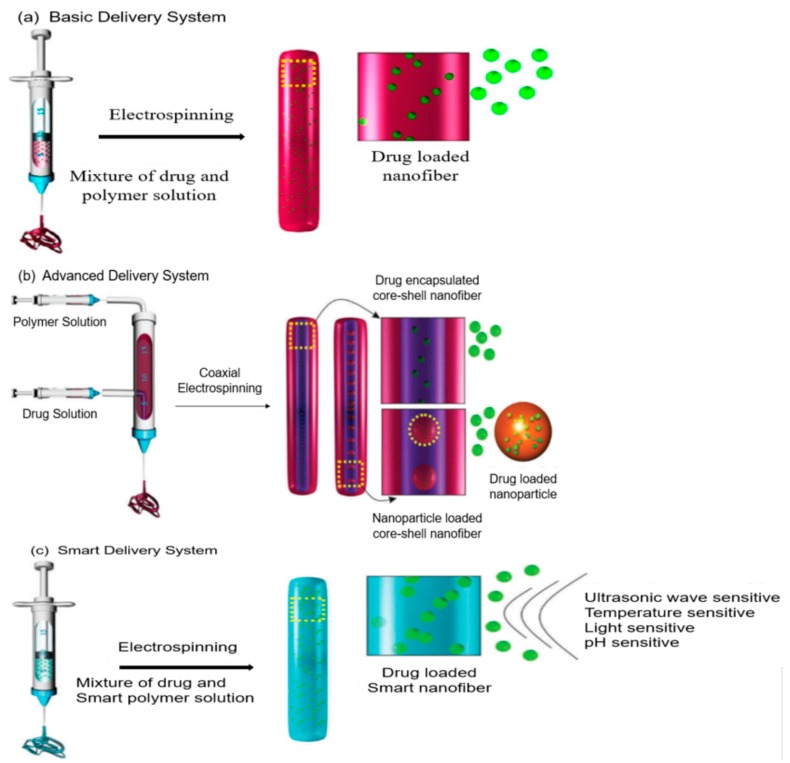
Drug loading strategies to nanofibers: (**a**) basic antibacterial delivery systems, (**b**) advanced antibacterial delivery systems (core–shell structure, nanoparticle decorated and multidrug loaded), and (**c**) smart delivery systems (stimuli responsive). (Reproduced from ref no [76] with permission from “Taylor and Francis Group”, Ref: AF/IEDC/P19/0117).

The amount of antibiotic release and the effectiveness of its therapeutic window for effective treatment and preclusion of bacterial infections depend on the antibiotic loading efficiency in the carriers. In this regard, nanofiber-based drug delivery systems have gained more consideration for controlled drug release. This decreased and sustained dosing frequency of antibiotics can result in fewer side effects, high patient compliance, minimized fluctuation of antibiotics level in the bloodstream and overcome dose-dependent toxicity. In the traditional strategy of nanofiber-based drug delivery systems, the electrospinning process fabricates antibiotic-loaded nanofiber scaffolds either by making a solution of drug/nanofiber before electrospinning or by loading the antibiotic onto the large surface of the nanofibers through physical or chemical modifications as shown in Figure 4a. Both of these strategies can proficiently control the antibiotic release. One such example would be aminopenicillin (AMX) loaded PEGylated PLGA electrospun nanofibers, where AMX powder was blended with PEG-modified PLGA, followed by the fabrication of nanofiber scaffolds (AMX/PLGA-PEG) via electrospinning. The in vitro release profile exerted about 51.5% and 90.7% of AMX release within 2 and 48 h respectively from 1% AMX/PLGA-PEG nanofiber, and the drug releasing activities continued for more than 10 days. Due to the improvement of hydrophilicity in the AMX/PLGA-PEG nanofiber, faster and prolonged drug release properties were achieved. In addition, 1% AMX/PLGA-PEG nanofiber scaffolds can inhibit approximately 95.9% growth of penicillin resistance gram-positive *Streptococcus aureus* at an AMX concentration of 60 μg/mL. The hemolysis and anticoagulant in vitro experiments revealed that they have great hemocompatibility and cytocompatibility [78]. In another study, RuO_4_ (ruthenium tetroxide) oxidized herringbone graphite carbon nanofibers (hGCNF) was prepared for surface labeling of antibiotics. The higher carboxylic acid groups of RuO_4_-Oxidized hGCNF served as the binding site of antibiotics during the acyl substitution reaction. Finally, as-synthesized nanofiber scaffolds were covalently functionalized with tobramycin, amikacin, and ciprofloxacillin and this antibiotic-labeled RuO_4_-oxidized hGCNF possessed magnificent antibacterial action versus *Pseudomonas aeruginosa* [79].

To control the antibacterial drug release pattern from the nanofiber-based carrier, another important method has recently been investigated in the fabrication of both multidrug loaded or core-shell electrospun nanofibers. Although both emulsion and coaxial electrospinning processes are generally used, coaxial electrospun core-shell nanofibers are more commonly used to regulate the sustained release profiles of the loaded drug (Figure 4b). Mainly, the drug molecules are encapsulated into the core region of this advanced core-shell structure of electrospun nanofibers whereas the outer shells act as a protector and also manage the release behaviors of the incorporated antibacterial drugs. Thus, the core portion and shell portion of a core-shell electrospun nanofiber scaffold can sustain the release profiles of the antibiotics. Due to the protective and biocompatible nature of the outer shell of antibacterial nanocarriers, the sensitive antimicrobial drug is more stable in blood plasma. This can further prolong its release characteristics and facilitate long term antibacterial activities without dose repetition. Apart from protecting the core region of the antibacterial drug, the shell membranes can function as an antibacterial agent that can generate bacterial resistance in biological environments. A shape memory polyurethane (core-shell) antibacterial nanofiber was synthesized with a coaxial electrospinning method in which a polycaprolactone-assisted shape memory polyurethane core was shelled with a pyridine presenting polyurethane. The results demonstrated that the developed core-shell nanofibers displayed enhanced antibacterial properties in both gram positive and gram negative bacteria [80]. On the other hand, the emulsion electrospinning process has also gained the attention of many researchers, in which the use of a single nozzle fabricated core-shell nanofiber scaffold is more simple and more advantageous over the coaxial electrospinning method. In addition, the production of biocompatible, prolonged release, and low toxic core-shell nanofibers with foaming free facilities allows researchers to employ emulsion electrospinning to develop nanofibers. For example, Chai and coworkers for the first time proposed antimicrobial core-shell nanofiber scaffolds that hold a colloidal particles emulsion by electrospinning. In their core-shell nanofibers (Van/OA-MION-PLA), the antibacterial drug vancomycin was in the core portion as a water phase and the polylactic acid solution served as the oil phase, while oleic acid coated magnetic iron oxide nanoparticles were used as an emulsifier. The in vitro vancomycin release study depicts that about 10% of burst redemption appeared within 5 h. Surprisingly, a prolonged release of vancomycin up to 25 days with approximately 57% of cumulative deliverance was observed from Van/OA-MION-PLA [81]. 

Furthermore, in the smart antibiotic delivery system, electrospun nanofiber scaffolds are commonly used to control the release of drugs in response to various biological parameters including pH factor, temperature or UV-light sensitivity (Figure 4c). Recently Fakhri and coworkers investigated the UV-light and pH-responsive photocatalytic activity of Tungsten disulfide (TDS), TDS-chitosan and TDS-polycaprolactone nanofibers with respect to the degradation of the antimicrobial Neomycin. The antibacterial drug Neomycin conjugated with TDS-chitosan and TDS-polycaprolactone nanofibers revealed a comparatively good decomposition rate of Neomycin as well as an antimicrobial performance at pH 3 [82]. In other research, the pH-dependent antibiotic release Eudragit nanofiber mesh was fabricated via a coaxial electrospinning process. The drug release study indicated that the release rate of Tetracycline from a Tetracycline-loaded Eudragit nanofiber mesh depended on both the physiological pH value and the molar ratio of pure Eudragit and Eudragit L100 in an Eudragit nanofiber mesh. At pH 6 the Tetracycline release was much faster from the as-fabricated nanofiber mesh, but at pH 2 the release rate was very slow. In addition, at the high molar ratio of the Eudragit nanofiber mesh, Tetracycline release was optimum in both pH values [83]. In comparison with other drug nanocarriers, nanofiber scaffolds present more potential applications for antibacterial drug delivery with sustained release properties. 

#### 5.1.3. NSAIDs

Nonsteroidal anti-inflammatory drugs (NSAIDS) distinguish themselves from steroids and are popular worldwide due to their anti-inflammatory actions. In addition, they are a frequently prescribed medication as they are widely known to have pain relieving, anti-pyretic, and blood clotting activities. NSAIDs usually reduce the synthesis of prostaglandins by inhibiting the actions of biological enzymes cyclooxygenase-1 and cyclooxygenase-2. However, these drugs are not free from side and adverse effects. The severe side effects associated with NSAIDs include GI ulcers, heart attack and kidney disorders [84,85]. 

The amount of poorly water-soluble NSAIDs is increasing daily in the pharmaceutical industry. To achieve the desired goal and for improving treatment with NSAIDs, electrospinning techniques have already been introduced in this sector. Ibuprofen is a class of NSAIDs, generally suggested for pain, fever, inflammation, migraines, arthritis, and painful menstruation. In one study, Potrč and co-workers showed that ibuprofen-loaded PCL nanofibers can enhance the dissolution rate of this loaded drug in a biological environment where almost 100% ibuprofen was released from as-synthesized nanofibers within 4 h [41]. Naproxen sodium is another cyclooxygenase inhibitor of the NSAIDs class which is used to treat inflammation including fever and rheumatoid arthritis, pain and menstrual cramps. Naproxen is also used in combination with sumatriptan succinate (a member of the triptans family) to treat migraines or with proton pump inhibitors (i.e., esomeprazole) to avoid NSAIDs-assisted acidity. A few years ago, naproxen and its salt (naproxen sodium) had been electrospun with various hydrophilic (chitosan, PVA and polyacrylic acid) and hydrophobic (PCL) polymers with very good drug loading capacity for achieving rapid onset of action, avoiding hepatic fast pass metabolism and readily accessible through the sublingual route of administration. All developed nanofibers provided the burst release of naproxen in which almost 90% of the released drug from all nanofiber scaffolds was dissolved within 10 min in the acceptor phase. In addition, under the same conditions, no significant differences had been observed between the release profiles of naproxen and its salted form (naproxen sodium). Somehow, the hydrophobic PCL fiber mat exhibited a very quick release of naproxen [86].

Electrospinning techniques are not only applicable to fast releasing, dissolving and complete absorbtion of drugs but also these methods are used for easy swallowing and taste masking of various drugs in case of oral administration. Meloxicam is such a type of NSAID, which is used to treat inflammation in rheumatoid arthritis and osteoarthritis as well as pain. However, its bitter taste, difficulty to swallow, and low bioavailability due to incomplete absorption after peroral administration limited its usage, especially in children [87]. To improve these demerits, an oral dissolving formulation was developed employing the electrospinning process. The incorporation of a cyclic oligosaccharide (cyclodextrin) into PVP nanofibers improved the physical stability of mats whereas the addition of sweeteners avoided the bitter taste of meloxicam. The meloxicam-encapsulated PVP/cyclodextrin fiber mats were a nanometer in size with suitable tensile strength. Interestingly, the nanofiber mats released 100% of the meloxicam in the artificial saliva pH 6.8 within 120 min of administration whereas the tablet and powder dosage forms of meloxicam (marketed product) released only 30% in the same environment. These studies indicate that the developed oral formulation was capable of increasing the solubility, disintegration time, release, bioavailability, and palatability of the encapsulated drug. However, the nanofiber mats disintegrated within 1 min on contact with mouth saliva and, after that, the disintegrated fiber mats continuously released the drug in the gastrointestinal tract [88]. Thus, electrospun nanofiber scaffolds have their own potential to overcome the problem related to keeping the nanofiber mats in the oral cavity for a long time to complete release of the drug. 

#### 5.1.4. Cardiovascular Agents

Disorders related to heart and blood vessels are categorized as various cardiovascular diseases such as stroke, heart attack, hypertension, cardiomyopathy, heart arrhythmia, carditis, aortic aneurysms, peripheral artery disease, thromboembolic disease, venous thrombosis and rheumatic, valvular and congenital heart disease [89]. Among these disorders, coronary artery diseases like angina and myocardial infarction are the most common cardiovascular disorders. Nicorandil is widely used against angina or angina pectoris due to its agonistic properties to both ATP-sensitive K+ channel and a polyatomic ion, nitrate, channel [90]. The low bioavailability and slow onset of activities and the major side effects including excess turnover rate and mucosal ulceration have limited the usage of nicorandil as an anti-anginal agent. To overcome these limitations, nicorandil had been electrospun with polymeric nanofibers composed of riboflavin, hyaluronic acid, and PVA to prepare a sublingual dosage for treating angina pectoris. It was expected that the presence of riboflavin in the nanofiber scaffolds would cure mucosal ulceration whereas hyaluronic acid would ensure the quick recovery of inflammation in damaged tissue by reducing the amount of pro-inflammatory cytokines. However, this nano-sized drug-loaded fiber mat was able to sustain the controlled release of nicorandil over a prolonged period of time. The pharmacokinetic study revealed the maintenance of a therapeutic level over a longer period of time and about 4 times more biological half-life of the developed formulation in comparison with marketed nicorandil. Moreover, no mucosal ulceration had been evidenced by the histopathological study at the site of administration for the developed formulation [91]. 

Carvedilol is another cardiovascular drug that binds and blocks both alpha and beta-adrenergic receptors in an attempt to treat congestive heart failure. Potrč et al. (2015) researched electrospun PCL nanofiber scaffolds as a delivery carrier for the oral administration of poorly water-soluble carvedilol. It was observed that the average size of a drug-loaded PCL nanofiber is directly proportional to the amount of loaded drug and the crystallinity of the carvedilol decreased after encapsulation into the PCL nanofiber. The encapsulated drug was partly molecularly interspersed in the PCL nanofiber and in the formation of dispersed nanocrystals to a certain extent. It had been reported that up to 77% of carvedilol was released from the PCL electrospun nanofibers within only 4 h which indicated a significant improvement of the dissolution rate of this poorly water-soluble drug [41]. Hence, electrostatic spinning is a novel nanotechnology-based strategy especially for improving the dissolution rate of water-insoluble drugs.

#### 5.1.5. Gastrointestinal Drugs

Drugs that are used against various gastrointestinal tract or gut or digestive system disorders or ailments to cure or prevent many severe symptoms of the esophagus, stomach, intestines (both small and large), rectum, and anus, are known as GIT drugs. GIT drugs include antidiarrheal, antiemetic, anti-ulcer agents, cathartics, cholagogues and choleretics, emetics, laxative, lipotropic agents, antibacterial and many other types which are frequently prescribed to control gastric juice, regulate gut motility, water flow and improve the digestion of patients [90]. Unfortunately, the inability of GIT drugs as a curative therapy may lead to surgery in the case of serious complications. Hence, finding a new method to improve the pharmacological action of GIT in human physiology is crucial, especially for serious diseases such as inflammatory bowel syndrome-assisted Crohn’s disease, gastroesophageal reflux disease or acid reflux disease, irritable bowel syndrome, and peptic ulcer disease that can lead to stomach cancer. 

Metoclopramide (4-amino-5-chloro-*N*-[2-(diethylamino) ethyl]-2-methoxybenzamide) is an antidopaminergic benzamide, pharmacologically used as a serotonin receptor agonist. Its inhibitory action on acetylcholinesterase exerts its prokinetic and anti-emetic effect due to its actions on contractility of colonic smooth muscle. Recently, Jaber and co-workers fabricated a core/shell nanofiber using PVA/PCL to load metoclopramide hydrochloride [92]. The release profile indicated an initial burst release of the loaded drug (about 55% of total release). The reason behind the initial burst release of metoclopramide may indicate the presence of micron or nano-sized pores in the PCL shell. 

Designing the control release behavior of hydrophilic molecules such as protein, peptide, nucleic acid or even a drug is a very difficult task. Tiwari et al. proposed a new method by controlling the partition release of two-layer fiber matrix using a core-shell electrospun strategy where the polymer will serve as an outer layer and the encapsulated drug will be in the core. The authors used metoclopramide to represent a hydrophilic drug and loaded it into various monolithic fibers (PCL, PLLA, PLGA, and PVA) as well as core-shell nanofibers such as PVA/PCL, PVA/PLLA, and PVA/PLGA to investigate the control release behavior of metoclopramide loaded as-synthesized nanofibers. The drug release profile data suggested that the controlled release of hydrophilic entities is possible by using core/shell nanofibers and by verifying the physicochemical properties of core/shell solutions. The result also showed a clear difference according to the release characteristics between the monolithic fibers, which are made of hydrophilic and hydrophobic polymers, and core/shell fibers using PCL, PLLA, and PLGA 80/20 shell polymers. The monolithic fibers cannot control the initial burst release of the hydrophilic drug metoclopramide hydrochloride but core/shell electrospun nanofibers can easily regulate the controlled release of incorporated drugs. Thus, electrospun nanofibers would be a promising gastrointestinal drug carrier to achieve controlled release behavior and protect sensitive drugs in a biological pH [93].

#### 5.1.6. Antihistamines

Antihistamine generally works by blocking the physiological activities of histamine, thus, antihistamines are used in the treatment of nasal congestion, sneezing, hives, seasonal hay fever and especially, to relieve the symptoms of various allergies such as dust allergy, cold allergy, allergic rhinitis, indoor and food allergies. For the time being, electrostatic nanofiber scaffolds are being used as a carrier of various antihistamine drugs. One experimental study was designed to incorporate the first-generation antihistamine (H1 receptor antagonist) chlorpheniramine maleate into glutinous rice starch combining polyvinyl alcohol (GRS/PVA) electrospun nanofibers to investigate a drug delivery carrier concept and control release properties of the nanofibers [94]. The hybrid nanofibers (GRS/PVA) offered a biphasic release of loaded-antihistamine in which 60% of initial release had taken place within first 10 min and reached the highest release at about 90% within 120 min of administration. The authors suggested this GRS/PVA nanofiber scaffold as a novel oral antihistamine drug carrier. 

Development of fast dissolving delivery systems of therapeutics (FDDST) is an excellent and unique concept especially for those patients who have difficulties swallowing pharmaceutical solid dosage forms. FDDST offers very fast-dissolving solid oral dosage forms that take a few minutes for absorption in the patient’s mouth. Thus, FDDST facilitates high drug bioavailability, therapeutic window and exemption of hepatic first-pass elimination. Previously, the electrospinning method was employed to design, develop and evaluate FDDST in which one study proved more than 80% of total ibuprofen release within 20 s [95] whereas another study showed the co-release of caffeine and vitamin B12 at about 100% and 40% respectively within 1 min [96]. However, Loratadine is another effective peripheral histamine H1 receptor inverse agonist and responsible for anti-allergic effects. Nevertheless, its low water solubility, bioavailability and rapid first-pass hepatic effect with mainly CYP3A4 and CYP2D6 (isoforms of cytochrome P450) metabolism systems reduce their usages. In this respect, electrospinning nanofiber-based Loratadine delivery would be the best solution. A few years ago, Akhgari et al. described the impact of few parameters such as the concentration of polymer and antihistamine, and the amount of feed ratio and the voltage on the first dissolving delivery systems for Loratadine. The authors prepared a Loratadine-encapsulated PVP nanofiber scaffold by way of the electrospinning technique and observed a lower feed ratio and low concentration of the polymeric solution with high voltage application produce nano-sized nanofibers with excellent uniformity. The results of this study showed that, to achieve quick solubility and release of antihistamine from as-prepared nanofibers, the fiber should be a nanometer in size and the antihistamine amount should be smaller [97].

Another example of an antihistamine is Diphenhydramine, a member of the ethanolamine class of histamine H1 receptor antagonists, which is mainly used to treat allergies, nausea, motion sickness, extrapyramidal symptoms and symptoms of a common cold by reversing the effects of histamine on capillaries. It is also used in parkinsonism due to its ability to act as a muscarinic acetylcholine receptor reverse agonist by crossing the blood-brain barrier. In addition, its usage as a sodium channel blocker introduced it as a local anesthetic [90]. However, to produce porous and fast-disintegrating nanofiber scaffolds targeting oral administration, diphenhydramine-incorporated nanofibers were directly electrospun onto a polymeric backing film of hydroxypropylmethylcellulose and glycerol [98]. The physicomechanical characterization data revealed the potency of nanofiber scaffolds in a nanofiber-based oral antihistamine delivery system with the following configurations: high encapsulation efficiency and very short disintegration times (12.8 s). This very short integration time may indicate the large surface area of PVA nanofibers, which were loaded with diphenhydramine. 

#### 5.1.7. Contraceptives

For the first time, a composite electrospun nanofiber was fabricated by free-surface electrospinning with various microscale geometries as a carrier of physicochemical diverse medicines including the contraceptive drug progestin levonorgestrel [99]. The as-fabricated PVA nanofibers were capable of encapsulating more than 80% of all matrix formulations except interwoven matrix where an artifact of the processing led to a calculated >150% encapsulation efficiencies for levonorgestrel. The authors checked the solid dispersion of levonorgestrel in the electrospun nanofibers by employing various differential scanning calorimetry analyses. After encapsulation of levonorgestrel, the thermograms of the PVA fabrics did not dramatically change even with high loading of levonorgestrel (17 wt.%). The in vitro release kinetics demonstrated slow and controlled release of levonorgestrel due to its highly hydrophobic nature, where levonorgestrel took 4 h to achieve 100% release from the as-synthesized composite microarchitectures. The idea is that these contraceptive-loaded nanofibers would be inserted into the vaginal mucosal environment to prevent unplanned pregnancy by releasing the appropriate dose of contraceptives into the local cells.

#### 5.1.8. Palliative Drugs

Palliative treatment—also known as comfort or supportive care—aimed to relieve, reduce or control symptoms, side effects and adverse effects such as pain and sickness at any stage of serious diseases, thus enhancing the life expectancy and comfort of a patient even during the last stages of an illness. 

Nagy et al. used lower molecular weight poly(vinyl alcohol) to fabricate the non-woven tissues of a nanofiber with a large surface area for developing an oral fast-dissolving dosage form by applying electrostatic spinning. After loading highly water-soluble Donepezil HCl (which is used to treat dementia-related Alzheimer’s disease) into as-prepared nanofibers, the diameter of the electrospun nanofiber was 100–300 nm even when the polymer-drug ratio was 33 w/w%. Interestingly, in comparison with the release rate of the commercially available Aricept tablet (brand name of Donepezil HCl), the drug-loaded nanofiber took less than 30 s to release whereas the Aricept tablet took ≥30 min. This might be due to the availability of the nanofiber’s large surface area which is directly proportional to the dissolution rate, corresponding to the Noyes and Whitney equation [100]. Hence, it can be said that organic solvent free electrospinning could be a promising option for manufacturing highly oral dissolving dosage forms for achieving instant drug release and quick onset action with high patient compliance, which is a basic requirement for palliative care policies.

#### 5.1.9. Miscellaneous

Among various miscellaneous drugs, caffeine is the most used agent worldwide that binds to adenosine receptors by mimicking natural adenosine, affecting various body functions. At present, electrospinning techniques are being widely used to fabricate caffeine loaded nanofibers as a FDDST facilitating a high surface area and high porosity of nanofibers that can lead to fast-wetting features of the nanofiber surface to ensure quick release properties of incorporated drugs. In these respects, drugs are usually encapsulated into nanofibers either in amorphous or nanocrystal format, ensuring high solubility, dissolution, rapid onset of action and bioavailability of loaded drugs. A study was designed by electrospinning using PVA as the drug nanocarriers of caffeine (CA) and riboflavin (RFN) in which the dissolution time and wetting time were 1.5 s and 4.5 s respectively for both PVA-CA and PVA-RFN. In addition, the release percentages of CA and RFN from PVA filament-forming nanofibers were 100 and 40 respectively within 1 min [96]. In another study, paracetamol and caffeine-loaded PVP electrospun nanofiber scaffolds were fabricated as a FDDST model with more than 90% drug loading capacity in which both loaded drugs remained intact even after electrospinning. This study indicated a high disintegration rate of the drug-loaded nanofiber scaffolds within 0.5 s whereas it took less than 150 s to reach 100% dissolution rate. The drug-loaded nanofiber matrix revealed its potency over the pure standard drugs (caffeine and paracetamol individually) [101]. Moreover, it was evidenced that electrostatic spinning has the potential to fabricate such nanofibers that can ensure the ultrafast drug delivery and dissolution of water-insoluble miscellaneous drugs even through the oral route of administration [102]. 

Discovering an effective and potential ophthalmic drug delivery system (ODDS) is still a challenge due to the quick elimination of ocular drugs like eye drops. Thus, rapid peroneal losses of eye drops, even with the high volume of administration resulting in low bioavailability and very short half-life of applied drugs and a lower amount of ophthalmic drugs (1–3% of total volume), can reach into intraocular tissues via penetration through the cornea. To address these problems, Gagandeep et al. (2014), developed a nanopatch using PVA and PCL via electrostatic spinning to load and deliver a combination of timolol maleate and dorzolamide hydrochloride as a model drug against glaucoma. As-developed drug-loaded nanopatches were 200–400 d·nm with almost 100% drug entrapment efficiency—no interaction has been found between polymers and encapsulated drugs. Furthermore, the in vitro drug release experiment alluded an initial burst release of incorporated drug from nanopatches followed by controlled release behavior for up to 24 h [103]. The overall results reveal the efficiency of electrospun nanopatches in ocular drug delivery. 

**Figure 5:**
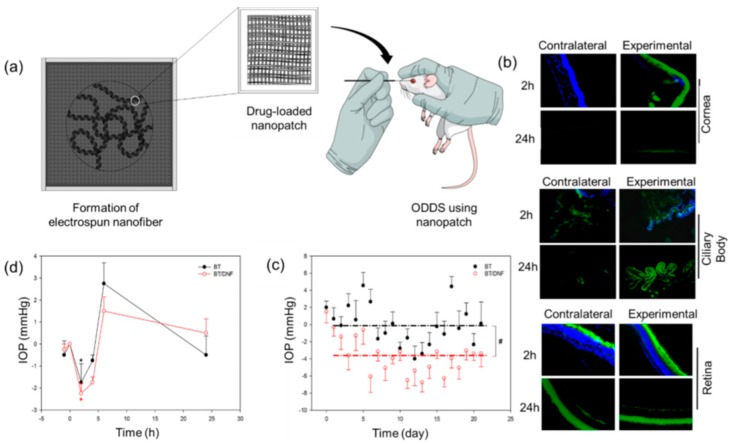
(**a**) An illustrative diagram of nanofiber-based ophthalmic drug delivery system; (**b**) Ocular deposition of dendrimer nanofibers (DNF). Brown Norway rats (BNR) (n = 3) received a DNF-FITC mat topically in the right eye (experimental eye), whereas the left eye received no treatment (contralateral eye); (**c**) In vivo single dose response. BNR (n = 4) received a single dose of brimonidine tartrate (BT) topically via saline eye drops or DNF; (**d**) In vivo 3-week daily dose response. Brown Norway rats (n = 4) received a daily dose of BT via saline eye drops or DNF for 3 weeks. (Here in, Figure 5b–d reproduced from ref. [58] with permission from ACS Publication, 2017).

Drug enrichment on the surface of the polymer is only possible if the drug is blended with the polymer solution before electrostatic spinning. This system facilitates an initial burst release of the loaded-drug resulting in rapid onset of action, but it also decreases the potential lifetime of delivery carriers. Hence, to obtain desired results such as sustained therapy, local and controlled release, enhanced activity and retrenchment of side and adverse effects, core-shell nanofibers would be the best option. One study was designed to load brimonidine tartrate (BT) into a first dissolving dendrimer nanofibers (DNF) against glaucoma (Figure 5a). The in vivo intraocular pressure (IOP) was evaluated as an efficacy parameter for both BT and BT-DNF (Figure 5b). As shown in Figure 5c, the single dose responses of BT and BT-DNF were almost similar to each other regarding the therapeutic effects. Nevertheless, after daily treatment with BT and BT-DNF over a period of 3 weeks, BT-DNF was more effective in comparison with BT for reducing IOP (Figure 5d) [58]. In another study, a novel method was established, combining both coaxial electrospinning and emulsion electrospinning to fabricate a core/shell fiber matrix. As-proposed PLGA coaxial-emulsion electrospun fibers were loaded with an anticonvulsant class of drug—Levetiracetam—and the aim was to implant them in the brain to treat various seizures in adult patients and children with epilepsy. The constant and linear release kinetics of the incorporated lower molecular weight drug Levetiracetam from fiber scaffolds was noticed during 480 h of the study [104].

### 5.2. DNA and RNA Delivery

The delivery of nucleic acid molecules such as DNA or interfering RNA, into a target cell to knockout/ knockdown mutated gene expressions, either by gene editing or impeding the mechanism of the mutated gene, has proven its novelty in the field of regenerative disease treatment. In this case, when a gene misbehaves in a protein, gene therapy is able to introduce the new copy of that specific gene for recuperating the function of that protein by adjusting the signal transduction pathway [105]. To date, the nucleic acid delivery system depends on the carriers and can be folded into two groups; a viral and a non-viral vector. As nucleic acid directly implanted into the cell does not work, researchers use vectors as their carrier. In viral vectors, useful viruses are genetically modified with a specific nucleic acid so that they can enter into the target cell and introduce new genetic material in the place of missing or faulty genes. However, viral vectors are very specific such as their delivery strategy depends on the type of tissues and gene, even they can carry a very small size of a gene that may sometime cause mutation. In comparison with a viral vector, non-viral vectors are more appreciable among scientists owing to their manageable toxicity, ability to carry various size of genes, large surface area, and large porosity [106]. Among all types of non-viral vectors, nanofiber scaffolds are widely used even though problems associated with electrospun nanofiber scaffolds, such as inappropriate nucleic acid encapsulation and transfection efficiency, are still unsolved. To overcome these limitations, several attempts have been introduced, for example, core/shell, surface modification, coating, encapsulation, incorporation, or interfacing electrostatic interaction, to protect the nucleic acid [107,108,109,110,111,112,113,114,115,116,117,118,119,120,121,122,123,124,125,126,127,128,129,130,131,132].

However, the encapsulation of a nucleic acid/polymer complex in the core of core/shell nanofiber scaffolds can protect the encapsulated DNA or interfering RNA from biological degradation and denaturation, as well as prolong their release up to several months by controlling it. For instance, non-knitted, membraneous nanofiber scaffolds were fabricated via the electrospinning procedure as a gene delivery carrier, containing plasmid DNA encapsulated with poly(lactide-co-glycolide) and poly(d,l-lactide)–poly(ethylene glycol) type biodegradable synthetic copolymers [107]. These promising nanofiber scaffolds can control the release behavior of plasmid DNA over 20 h of the study, whereas the burst release of plasmid DNA appeared within 2 h of the study. In addition, the cumulative release profile indicated up to 80% of plasmid DNA was released from nanofiber scaffolds. The released plasmid DNA had a high cellular transfection efficiency with specific protein encoding properties. In another study, various fiber mesh scaffolds were designed and prepared through the coaxial electrospinning method containing the plasmid DNA and non-viral gene delivery vector poly (ethyleneimine)-hyaluronic acid within the core and sheath polymer of poly(ethylene) glycol and poly(ε-caprolactone), respectively. The cumulative releases of plasmid DNA and the non-viral gene delivery vector from the core and sheath of fiber mesh scaffolds over a time period of almost 2 months showed a dramatic increase in transfection efficiency compared to the control group [108]. Though variously modified nanofiber scaffolds are used today to protect and encapsulate plasmid DNA-like genetic materials, the blending of plasmid DNA with an electrospun solution does not facilitate proper encapsulation and protection of biotherapeutics. In this case, the incorporated plasmid DNA is not uniformly distributed throughout the nanofiber scaffolds, which may hamper their release profile. Hence, surface modification with a cationic polymer could solve these issues. Kim et al. designed DNA-loaded surface modified nanofibers for epidermal gene delivery with a matrix metalloproteinases (MMPs) responsive control release behavior. Here, a MMPs-cleavable linker was used for surface modification of as-synthesized poly(ethylene) glycol/poly(ε-caprolactone) nanofiber meshes with linear polyethyleneimine (LPEI) so that the external MMPs can breakdown the MMPs-linker and facilitate the MMPs-responsive control release of the DNA-loaded polymer [119]. The release studies revealed that more than 80% DNA and almost 80% LPEI can release from the proposed nanofiber mashes over a 72 h time period whereas the transfection efficiency mainly depended on the charge ratio between DNA and LPEI rather than the amount of release. 

To date, various natural and synthetic polymers have been used to fabricate electrospun nanofibers in order to allow the successful delivery of genetic materials to the target site. In addition, the hybrid blending of natural and synthetic polymers has also been introduced for the same purpose. In Table 1 we illustrate the usages, designs and advances of various nanofibers as delivery platforms for DNA and RNA, respectively.

Nanofiber-based siRNA delivery is also in the spotlight due to its promising mechanism for silencing specific gene expression, targeting various diseases. Those genes have the ability to develop genetic mutations and block the secretion of inhibitory factors resulting in excess cell proliferation that may lead to cancer. To date, various electrospun nanofiber scaffolds have been used as a siRNA nanocarrier to deliver siRNA into the physiological system. Among them, PCL is the most used nanofiber scaffold that facilitates high siRNA loading efficiency, local delivery of siRNA, control release behavior, higher cellular transfection, manageable toxicity and maximum gene silencing properties [114].

However, the hydrophobic nature and slow degradation properties of PCL are responsible for the slow release properties of encapsulated siRNA [110,121]. To overcome this issue, surface modification, the formation of the block polymer and the polydopamine coating of PCL types strategies are taken into consideration which are briefly described in Table 1. Peptide-based nanofiber scaffolds have also been introduced as an siRNA nanocarrier targeting neurodegenerative disease. Their unique characteristics facilitate targetwise siRNA release and accumulation, a high residence period of siRNA in the brain region, a promising gene silencing profile, and ensure genetic intervention [123]. In addition, for the first time, scientists proposed a zein nanofiber-inspired siRNA delivery system that ensures proper siRNA encapsulation and the sufficient release of siRNA, high loading efficiency, cellular attachment, and transfection of siRNA. This zein-based electrospun nanofiber successfully preserves the efficiency of siRNA [113]. Various nanoparticles, except for PCL, such as PEG (polyethylene glycol), PCLEEP (poly(ε-caprolactone-co-ethyl ethylene phosphate)), ZnGa2O4:Cr (chromium-doped zinc gallate), P-G3A3KRK (Palmitoyl-GGGAAAKRK peptide), Zein, PECL (poly(ε-caprolactone)), PEG-b-P4VP ((Poly(ethylene glycol)-*b*-poly(4-vinylpyridine)), PLGA (poly(lactide-co-glycolide)), LPEI (linear polyethyleneimine), ELP (elastin-like polypeptides), PDLLA (poly(d,l-lactide)), and many more are used as a nucleic acid carrier [111,112,113,120,122,123,125] and are summarized in Table 1.

### 5.3. Growth Factor

**Figure 6:**
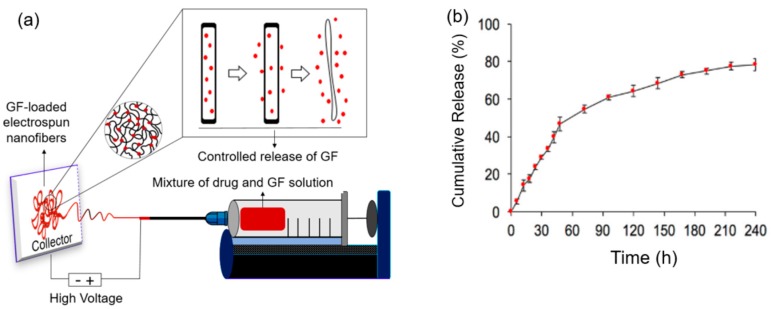
Schematic of the synthesis of growth factor loaded electrospun nanofiber with the controlled release properties (**a**), and VEGF (vascular endothelial growth factor) release profile with respect to time from PCL nanofibers (Reproduced from ref no [123] with permission from ACS Publications, 2018) (**b**).

Growth factors are naturally occurring endogenous signaling molecules (i.e., proteins or steroid hormones) which are efficient for inciting cellular development, differentiation, regeneration and proliferation by binding to cellular receptors. Recently, marked progress has been made in preparing several types of ultrathin electrospun nanofiber scaffolds to successfully load and deliver several growth factors (Figure 6a) as well as regenerative medicines, owing to the unique characteristics of nanofibers such as large surface area, porous formation, high loading capacity, easy access, and cost-effectiveness [133]. Notably, the large surface area to volume ratio of electrospun nanofibers facilitates cell adhesion, loading, storage and release of growth factors [134]. Those parameters are required for guiding cellular behaviors and transmitting signals that regulate proliferation, differentiation, metabolism, and apoptosis of cells, including extracellular matrix deposition in tissue engineering. 

The quick inactivation and very short biological half-life of growth factors are the most important factors hindering their effectual delivery. In addition, it has been noted that the growth factors’ carrier should have slow and sequential releasing properties. The establishment of growth factors into nanofiber scaffolds play very crucial roles in repairing and regenerating damaged tissues by mimicking signal transduction from cell to cell, or from cell to its extracellular matrix [135]. Either electrospinning procedures alone (for example, bending, emulsion, and coaxial electrospinning) or in combination with other traditional methods such as hydrogel (electrospinning) are ranked at the top among all nanofiber fabrication techniques for their controlled and sustained release properties. On the other hand, surface modified nanofibers promote high loading efficiency, protection of incorporated growth factors and the binding of growth factors to cell receptors. Those surface modified nanofibers exhibit biochemical and morphological uniformity to natural tissues. 

Recently, the polysaccharide matrix is being used as a superior nanofiber material to fabricate a growth factor carrier owing to their low toxicity, biocompatibility, and extracellular matrix mimicking capability. However, introducing the sulfation state to the alginate scaffolds ensures the appointed binding and loading of growth factor into the polymer backbone, as well as maintenance of its control release properties. For instance, Mohammadi S, et al. fabricated electrospun scaffolds by blending alginate sulfate and polyvinyl alcohol to load and deliver transforming growth factor-beta1 (TGF-β1) for tissue engineering applications. For the first time, this research revealed that more successful binding, encapsulation and sustain release of heparin-like proteins from electrospun scaffolds could be achieved by the addition of an affinity site to the alginate [134]. A part of mammalian polysaccharides—glycosaminoglycans (heparin-like substance)—would be another example of an excellent nanofiber matrix because of its efficiency in conjugating with GFs by successfully interacting with its (GFs) anionic sulfated backbones and mimicking a pathway similar to ECM.

Usually, the traditional nanofiber scaffolds-based GFs delivery methods such as dip coating and covalent binding are associated with low entrapment efficiency and primary burst release properties. Currently, researchers are looking for new, innovative and stable strategies by focusing on controlled and sustained release properties. To control the release properties of encapsulated GFs, the usage of gelatin particles in nanofiber fabrication is becoming popular. Generally, the in vitro degradation time of gelatin particles is about 240 h, so researchers tend to use gelatin particle-mediated nanofiber scaffolds as a promising GF nanocarrier. The basic advantages of using gelatin particles are their slow degradation and controlled release properties in a biological environment because of introducing cross-linking treatment during fabrication. An example would be the work of Jiang and co-workers who prepared PCL fibrous scaffolds containing vascular endothelial growth factor (VEGF)-incorporated gelatin particles which were able to exhibit both diffusion and degradation mediated release for a long period of time (Figure 6b), differentiate mesenchymal stem cells from endothelial cells, sustain the durability of the tubular composition, and form new blood vessels among endothelial cells [136]. The integration of various sustained release characteristics within nanofiber scaffolds protects the encapsulated GFs from biological degradation, generates cellular signal transduction as well as repairs and regenerates damage. 

Biocompatible and biodegradable polymeric nanoparticles and biomimetic hydrogel have gained more attention for encapsulating various types of growth factors and confirming their controlled and prolonged releasing [133]. VEGF and bone morphogenetic protein 2 (BMP2) were incorporated and injected to improve the angiogenesis and regeneration of bone in rabbits [133]. Another preclinical study suggested that VEGF-loaded and hMSC (human bone marrow stroma cells)-seeded PDLA nanofiber scaffolds are able to regenerate blood vessels and enhance bone growth [137]. Interestingly, to achieve dual actions from two separate GFs at a time, core/shell nanofibers or emulsion electrospinning methods are widely used in tissue engineering applications. Liu et al. prepared bicomponent scaffolds with the emulsion electrospinning method for delivering two GFs at the same time in nerve tissue engineering. In this study, the nerve growth factor was encapsulated in poly(d,l-lactic acid) (PDLLA) nanofiber scaffolds whereas the glial cell line-derived neurotrophic factor was incorporated in poly(lactic-co-glycolic acid) (PLGA) electrospun scaffolds [138]. This dual factor core/shell electrospun nanofiber scaffold not only revealed its sustained release and degradation-dependent weight loss properties over 42 days but also ensured the high entrapment efficiency and the intact bioactivity of GFs. Apart from degradation-based controlled release behavior of electrospun scaffolds, providing the affinity sites and/or covalent binding of heparin to the surface of nanofibers is also established. The covalent binding of heparin-like proteoglycans on the surface of the nanofibers preserves the biological integrity of GFs and promotes the sustained release properties of the scaffolds. The aminated PLGA was immobilized with VEGF in the presence of heparin via EDC/NHS coupling to facilitate the sustained release properties of the encapsulated growth factor [139]. The presence of hydroxyl (–OH) and carboxylic acid (–COOH) groups on the surface of the heparin simplifies its modification with both GFs and nanofiber scaffolds. Heparin also increased the binding capacity of VEGF to VEGF receptors on the cell surface.

The time period of sustained release of GFs from its carrier should be proportional to the time period of GFs capability to preserve the nanofiber scaffolds; therefore, this important factor is considered during the design of nanofiber-mediated GFs delivery. Employing several electrospinning techniques, the preservation of GFs’ bioactivity is achieved for the proliferation and migration of specific cells. The heterogeneous electrospun matrix made of biocompatible poly(l-lactide-co-caprolactone) and poly(2-ethyl-2-oxazoline) was able to protect the bioactivity of dual angiogenic basic fibroblast growth factor and vascular endothelial growth factor during and after their physical blending. It is also capable of mimicking the topography and chemical cues of native cardiac tissue structures [140]. The structural integrity and bioactivity of encapsulated GFs remained the same over the total period of sustained release in comparison with the pure GFs. In another study, for the first time, Naskar et al. reported the anteriority of using natural polymer silk protein fibroin reinforced synthetic carbon nanofiber scaffolds for preserving bioactivity and controlling the sustained release kinetics of BMP-2 and TGF-β1 GFs over a prolonged period of 50 days [141]. The primary attachment of GFs and the regeneration, excess proliferation, and differentiation of both osteoblastic cells and MSC proved the structural integrity of encapsulated GFs in the nanofiber scaffolds. Moreover, a few linear polysaccharides (i.e., chitosan) are very popular for encapsulating GFs (transforming growth factor beta-1). The major challenge associated with the polysaccharide-based GFs delivery system is their short biological half-life. Hence, they are further composited with nanofiber scaffolds in order to improve the long term presence of GFs in stem cells through maintaining their sustained release from electrospun scaffolds [142]. Previously reported data proved the efficiency of electrospinning methods to load and achieve the sustained release properties of two different GFs at the same time in tissue engineering applications, but now it is also possible to accomplish the distinct release kinetics and spatiotemporal delivery of two different GFs from the same scaffolds with various layered configurations by applying several electrospinning techniques. The combination of emulsion, sequential, high speed and double-sourced double power electrospinning were employed to fabricate tri and tetra layered nanofiber scaffolds using poly(lactic-co-glycolic acid) and poly(d,l-lactic acid) [135]. This glial cell line-derived growth factor and nerve growth factor incorporated multilayer electrospun nanofibers was able to maintain various release actions of these GFs due to aligned fiber coating in each layer with a controlled thickness. The cumulative release studies of encapsulated-GFs indicated distinct release properties from different sites of tri-layered and tetra-layered nanofiber scaffolds over a period of 42 days.

## 6. Conclusions and Future Outlook

Electrospinning is a promising technology for manufacturing nanofibers from the laboratory to industrial level. Nanomaterials possess a large surface area and enhanced porosity, which are advantages for drug delivery and many other biomedical applications. Nanofibers can be formed using three different techniques: electrospinning, self-assembly, and phase separation. Electrospinning is the most widely used technique because of its promising results. Electrospun nanofibers have attracted much attention due to their biocompatibility, adhesiveness, sterile nature and their efficiency in diverse applications. Currently, nanofibers are considered suitable candidates for scaffold materials, wound dressing materials, drug delivery systems, filtration membranes, catalysts for reduction and so on. Nanofibers are used for medical applications, batteries and fuel cells as a new material.

Though the application of nanofibers is versatile, still more development and research is required for the proper use of nanofibers, especially for biomedical therapeutic delivery. The application of nanofibers in drug delivery, medical implants or tissue engineering is still very limited and most of the materials cannot reach the stage of clinical trials. A thorough investigation on the scaling up of nanofiber technology is required, to make it widely available. 

## Figures and Tables

**Table 1:** Electrospun nanofiber-based DNA/RNA delivery.

Design Strategy	Therapeutics	Polymer	Facilities	Ref
Core-Shell	pDNA (pCMVb encoding β-galactosidase)	PLA-PEG-PLA, PLGA	Prevent DNA degradation. Protect the bioactivity of DNA during electrospinning. Controlled and sustained release of DNA. High Transfection efficiency.	[126]
	pDNA encoding pCMV-EGFP	PEI-HA, PEG, PECL	Extended-release properties (>120 d). High transfection efficiency. Controllable release kinetics. High Expression of EGFP.	[108]
	AV encoding-gene for GFP	PECL, PEG	Controlled and porogen-assisted release behavior. High and localized transgene expression. Very low proliferation rate. Controlled virus exposure. The lower level of IL-1β, TNF-α, and IFN-α.	[127]
	pDNA (pEGFP-N_2_ encoding-GFP)	PELA	Sustained the controlled release. High Transfection efficiency. Promising cell viability. Highly controlled spatiotemporal gene expression. Promote tissue regeneration.	[128]
	pDNA (pbFGF and pVEGF)	PELA, PEI	Sustain release properties (>28 d). Enhance cell attachment, viability and protein expression. Higher cellular transfection. Ensure extracellular secretion of collagen IV and laminin. Downregulate inflammation. Produce microvessels and mature blood vessels.	[129]
	Circular pDNAs; [pcDNA3.1/myc-His− (A), and pcDNA3.1/myc-His(−)/lacZ]	PEG-b-P4VP	Offers tetrasome like pathway for DNA delivery. Ensure highly controllable kinetics.	[130]
Coating, Encapsulation, Incorporation, or Interfacing	pDNA (PT7T3D-PacI containing BMP-2	PLGA/Hap, Chitosan	Stable bioactivity of BMP-2 plasmid. Enhance Cell attachment ability. Low toxicity and immunological effects. Controlled release profile and bone regeneration capability. High DNA transfection efficiency.	[131,132]
	AAV r3.45 (cDNA encoding-GFP/CMVP)	ELP, PECL	Highly efficient cellular transduction. Influencing cell viability. Temperature-sensitive release properties.	[109]
	GAPDH siRNA	PCL, PEG	Control release of siRNA (>28 d). High cellular transfection. Up to 81% of GAPDH gene silencing efficiency. Local delivery of siRNA. Low toxicity. Tissue regeneration.	[110]
	Silencer^®^ GAPDH siRNA	PCLEEP with transfection reagent	Sustained the controlled release of siRNA (>28 d). More than 97% release kinetics profile. Prominently induced the desired gene silencing.	[111]
	EGFP-specific siRNA duplexes	PLGA, Chitosan	Excellent release properties. Prolonged and efficient gene silencing	[112]
	GADPH siRNA	Zein	Offers proper encapsulation with intact bioactivity of siRNA. High loading efficiency. Ensure the sufficient release of siRNA. High cellular attachment and transfection efficiency. More significant gene knockdown.	[113]
	REST siRNA (siREST)	PCL	Controlled REST knockdown of specific neuronal cells. Prominently induced the desired gene silencing. Generate functional neurons as therapeutics.	[114]
Surface Modification	pDNA (pGL3 encoding luciferase)	PLA, PEI	High cultivation period (>5 d). Large surface-to-volume ratio and highly flexible. Manageable transgene expression.	[115]
	pDNA (Plasmid EGFP-N2)	HAp, PDLLA	Facilitate > 95% of the accumulated release (>14 d). High cell viability and density. High GFP expression.	[116]
	pDNA (pEGFP-N1)	PEG/PECL with LPEI	High transfection efficiency. MMp-responsive control release.	[119]
	siRNA	PEG-PECL with LPEI	MMP-2 responsive siRNA release. Excellence gene silencing effects. Facilitates neo-collagen accumulation at the wound sites. Wound recovery can restore to normal levels. Improve the prognosis of diabetic ulcers. Low toxicity.	[117]
	REST siRNA (siREST)	PECL	High loading efficiency. Prevent initial burst release. Enhance gene knockdown efficiency. Increase neuronal markers expressions. Reduce glial cell commitment.	[118]
	hTERT siRNA	ZnGa_2_O_4_: Cr	Increase the siRNA concentration in-situ. High cellular transfection efficiency. Enhance gene silencing effects.	[120]
	REST siRNA (siREST, s11932)	PCL	Low toxic. Prominently induced the desired gene silencing.	[121]
	Silencer^®^ COLA1 siRNA (siCOLA1)	PCLEEP	Prolonged the availability of siRNA (≥30 d). High cellular transfection efficiency. Ensure genetic intervention.	[122]
Electrostatic Interaction	pDNA (pCMVβ encoding β-galactosidase)	PLGA, PLA-PEG	Up to 80% of intact released of pDNA (>20 d). Capable of cell transfection and bioactivity. Able of tissue regeneration. Capable to deliver a combination of genes in a controllable sequence.	[107]
	siRNA	Palmitoyl-GGGAAAKRK peptide (P-G3A3KRK)	Provide localized targeted gene delivery. Ensure intracellular uptake. Enhance siRNA residence time in the brain region. Successfully downregulate the specific gene expression and increase the apoptosis rate. Ensure genetic intervention.	[123]
DCEF	pDNA (pEGFP-C3)	Alginate/PCL	Improve gene immobilization. Enhance cell adhesion, viability, and proliferation. High Transfection efficiency. Biocompatible. Tissue Regeneration.	[124]
Controlling fibrous capsule formation	Silencer^®^ COL1A1 siRNA (siCOL1A1)	PCLEEP	Localized and sustained delivery (>28 d). High loading efficiency. Downregulates COL1A1 both in vitro and in vivo. Enhance cellular uptake. Prominently induced the desired gene silencing.	[125]

pDNA: plasmid DNA, PEG: polyethylene glycol, PLA: poly(lactide), PDLLA: poly(d,l-lactide), PLGA: poly(lactide-co-glycolide), PEI: poly (ethyleneimine), HA: hyaluronic acid, PCL: poly(caprolactone), PECL: poly(ε-caprolactone), PELA: poly(dl-lactide)–poly(ethylene glycol), Hap: hydroxyapetite, P4VP: poly(4-vinyl pyridine), LPEI: linear polyethyleneimine, ELP: elastin-like polypeptides, siRNA: short interfering RNA, PEG: polyethylene glycol, PLGA: poly(lactide-co-glycolide), PCL: poly(caprolactone), PECL: poly(ε-caprolactone), LPEI: linear polyethyleneimine, PCLEEP: poly(ε-caprolactone-co-ethyl ethylene phosphate), ZnGa_2_O_4_:Cr: chromium-doped zinc gallate.
